# Recent advances in transthyretin amyloidosis therapy

**DOI:** 10.1186/2047-9158-3-19

**Published:** 2014-09-13

**Authors:** Mitsuharu Ueda, Yukio Ando

**Affiliations:** 1 Department of Diagnostic Medicine, Graduate School of Medical Sciences, Kumamoto University, 1-1-1 Honjo, 860-0811 Kumamoto, Japan; 2 Department of Neurology, Graduate School of Medical Sciences, Kumamoto University, 1-1-1 Honjo, 860-0811 Kumamoto, Japan

**Keywords:** Transthyretin, Amyloidosis, Familial amyloidotic polyneuropathy, Senile systemic amyloidosis, Immunotherapy, Gene therapy

## Abstract

Mutant (MT) forms of transthyretin (TTR) cause the most common type of autosomal-dominant hereditary systemic amyloidosis—familial amyloidotic polyneuropathy (FAP). Until 20 years ago, FAP was thought to be an endemic disease, but FAP is known to occur worldwide. To date, more than 130 mutations in the TTR gene have been reported. Genotype-phenotype correlations are seen in FAP, and some variation in clinical presentation is often observed in individual kindreds with the same mutation and even among family members. Of the pathogenic TTR mutations, Val30Met was the first to be identified and is the most frequent known mutation found throughout the world. Studies of patients with FAP amyloidogenic TTR (ATTR) Val30Met documented sensorimotor polyneuropathy, autonomic dysfunction, heart and kidney failure, gastrointestinal tract (GI) disorders, and other symptoms leading to death, usually within 10 years of the onset of disease. Diagnosis is sometimes delayed, especially in patients without a clear family history and typical clinical manifestations, since diagnosis requires various studies and techniques such as histopathology, genetic testing, and mass spectrometry. For treatment of FAP, liver transplantation (LT) reportedly halts the progression of clinical manifestations. Exchange of an FAP patient’s diseased liver with a healthy liver causes MT TTR in the body to be replaced by wild-type (WT) TTR. Although clinical evaluations indicated that progression of other clinical symptoms such as peripheral neuropathy, GI symptoms, and renal involvement usually halted after LT in FAP ATTR Val30Met patients, recent studies suggested that LT failed to prevent progression of cardiac amyloidosis in FAP ATTR Val30Met patients after LT, with this failure reportedly being due to continued formation of amyloid that derived mainly from WT TTR secreted from the transplanted non-mutant liver graft. In recent years, many therapeutic strategies have been proposed, and several ongoing therapeutic trials involve, for example, stabilizers of TTR tetramers (tafamidis and diflunisal) and gene therapies to suppress TTR expression (antisense methods and use of small interfering RNAs). These novel therapies may prove to prevent progression of FAP.

## Introduction

Mutant (MT) forms of transthyretin (TTR) cause the most common type of autosomal-dominant hereditary systemic amyloidosis—familial amyloidotic polyneuropathy (FAP) [1-3]. In recent years, many therapeutic strategies have been proposed, and several therapeutic trials for FAP are ongoing [4,5]. Here, we review clinical presentation, pathogenesis, diagnostic purpose, and recent advances in the treatment of this disease.

### Amyloidosis and TTR

Amyloidosis is a protein conformational disorder characterized by extracellular accumulation of amyloid fibrils derived from various proteins [6-8]. Thus far, 30 distinct protein precursors of amyloid fibrils have been identified as causing different kinds of amyloidosis [9-12]. Depending on the type of amyloidosis, various factors can be responsible for protein aggregation.

**Figure 1:**
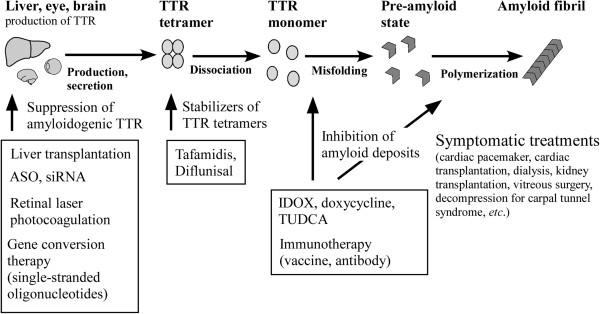
TTR amyloid formation and therapies for FAP.

TTR, a major amyloidogenic protein, is mainly synthesized in the liver [13] but also in the choroid plexuses of the brain [14], retinal pigment epithelial (RPE) cells of the eye [15], and α-cells of pancreatic islets [16]. TTR forms a homotetramer that has a dimer-of-dimers configuration in the bloodstream and that acts as a plasma transport protein for thyroid hormone and retinol-binding protein with vitamin A [17]. The plasma TTR concentration is reduced in conditions involving inflammation and protein malnutrition [18]. TTR causes two kinds of amyloidotic diseases. One is a hereditary systemic amyloidosis, FAP, which is induced by MT TTR [1-3]. The other type is senile systemic amyloidosis (SSA), which is an aging-related sporadic systemic amyloidosis that is induced by wild-type (WT) TTR [19,20]. Destabilization of TTR tetramers is widely believed to be a critical step in TTR amyloid formation (Figure 1) [21].

### FAP

Until 20 years ago, FAP was believed to be a disease that was restricted to an endemic presence in those specific areas. However, progress in biochemical and molecular genetic analyses resulted in an understanding that this disease now occurs worldwide. To date, more than 130 mutations in the TTR gene have been reported [2,22]. Although 15 TTR mutations are nonamyloidogenic, other TTR mutations induce systemic amyloidosis, which can be classified into several phenotypes including peripheral neuropathy dominant type, commonly called FAP; cardiomyopathy dominant type, also known as familial amyloidotic cardiomyopathy; vitreous opacity dominant type, which is thought to be derived mainly from TTR synthesized from RPE cells; and leptomeningeal amyloidosis dominant type, which is believed to be derived mainly from the choroid plexuses of the brain and causes central nervous symptoms. Hereditary TTR amyloidosis manifests genotype-phenotype correlations [2,23,24], and some variation in clinical presentation is often observed in individual kindreds with the same mutation and even among family members. Of the pathogenic TTR mutations, Val30Met was the first to be identified and is the best known mutation found throughout the world, although the reason for this distribution is not known.

**Figure 2:**
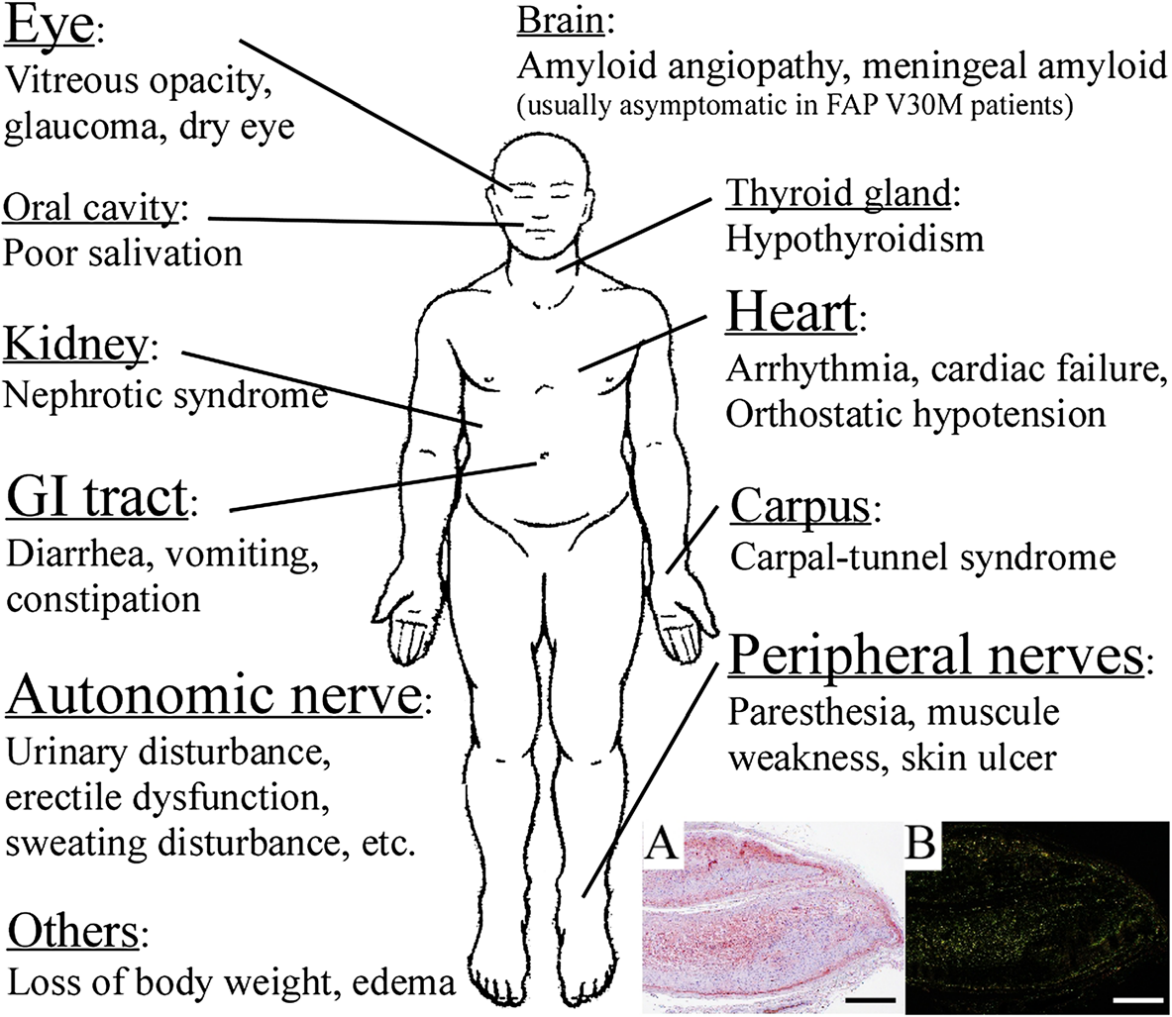
**Clinical manifestations in FAP ATTR Val30Met patients. (A, B)** Amyloid deposits in the sciatic nerve. **(A)** Congo red staining. **(B)** Polarized light. Scale bars: 500 μm.

**Figure 3:**
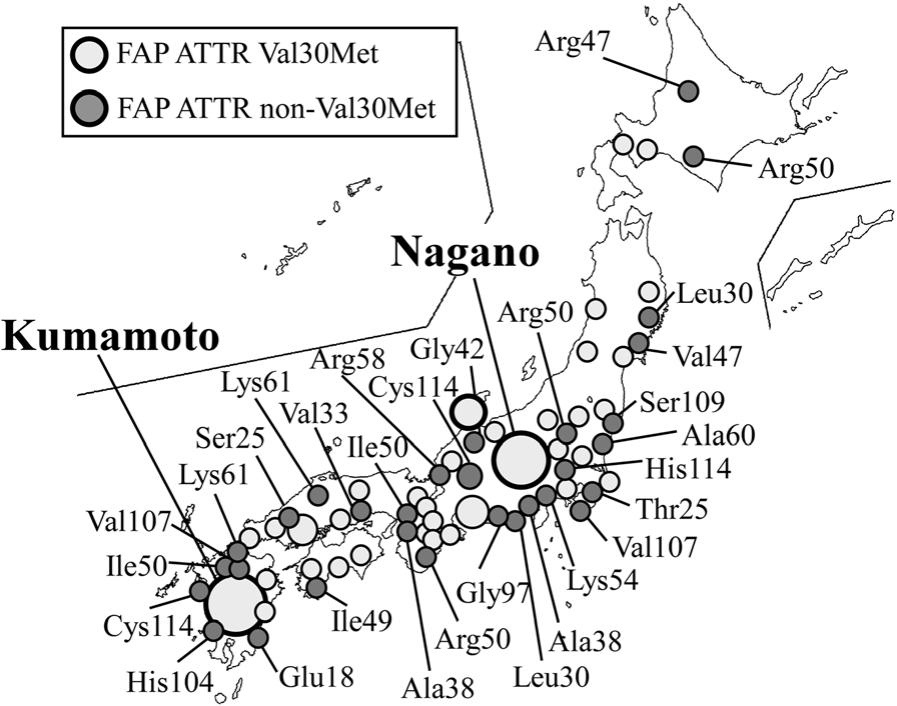
Distribution of hereditary TTR amyloidosis in Japan.

In Japan, Araki *et al*. first reported a group of patients with FAP ATTR Val30Met in Kumamoto [22]. Sensorimotor polyneuropathy, autonomic dysfunction, heart and kidney failure, gastrointestinal (GI) tract disorders, and other symptoms (Figure 2) that led to death, usually within 10 years of the onset of disease, have been documented in patients with FAP ATTR Val30Met [1]. In addition to the two endemic foci that were identified in Japan, many other FAP kindreds with TTR Val30Met and other mutations were found in Japan (Figure 3). That phenotypic differences exist among patients with the same Val30Met mutation and depend on geographic origin is well known [3,25-27]. Families originating from Portugal and two endemic areas in Japan (Arao city in Kumamoto Prefecture and Ogawa village in Nagano Prefecture) usually have early-onset and high-penetrance FAP, whereas other Japanese kindreds and Swedish families evidence late-onset and low-penetrance FAP [3,25-27]. Recent studies reported that certain Swedish patients with late-onset FAP ATTR Val30Met have amyloid deposits containing truncated TTR [28-31], which is usually found in SSA, a sporadic form of TTR amyloidosis [28,32]. Morphological ultrastructural studies showed that amyloid fibrils in those cases were tightly packed, haphazardly arranged, and fairly short compared with fibrils from other FAP ATTR Val30Met patients, usually with early-onset disease, who did not have truncated TTR in amyloid deposits [28]. The specific functional and pathological roles of truncated TTR remain to be determined, however.

### SSA

SSA, in which WT TTR forms amyloid deposits in various tissues, is an age-related nonhereditary systemic amyloidosis and affects mainly cardiac functions in elderly people [19,20,33-35]. Postmortem studies demonstrated that the prevalence of SSA was 12–25% in patients older than 80 years [19,20]. Furthermore, recent studies determined that WT TTR amyloid may also cause several other disorders and conditions [12], such as radiculomyelopathy [36], tongue necrosis [37], hematuria [38], nodular amyloid deposits in the lung [39], and frequent amyloid deposition in ligaments and tendons such as carpal tunnel and spinal ligaments [40,41], to a degree not previously known. In addition to full-length WT TTR, truncated C-terminal WT TTR fragments starting at positions 46–52 are usually detected in amyloid deposits obtained from SSA patients [32,35].

### Diagnosis

**Figure 4:**
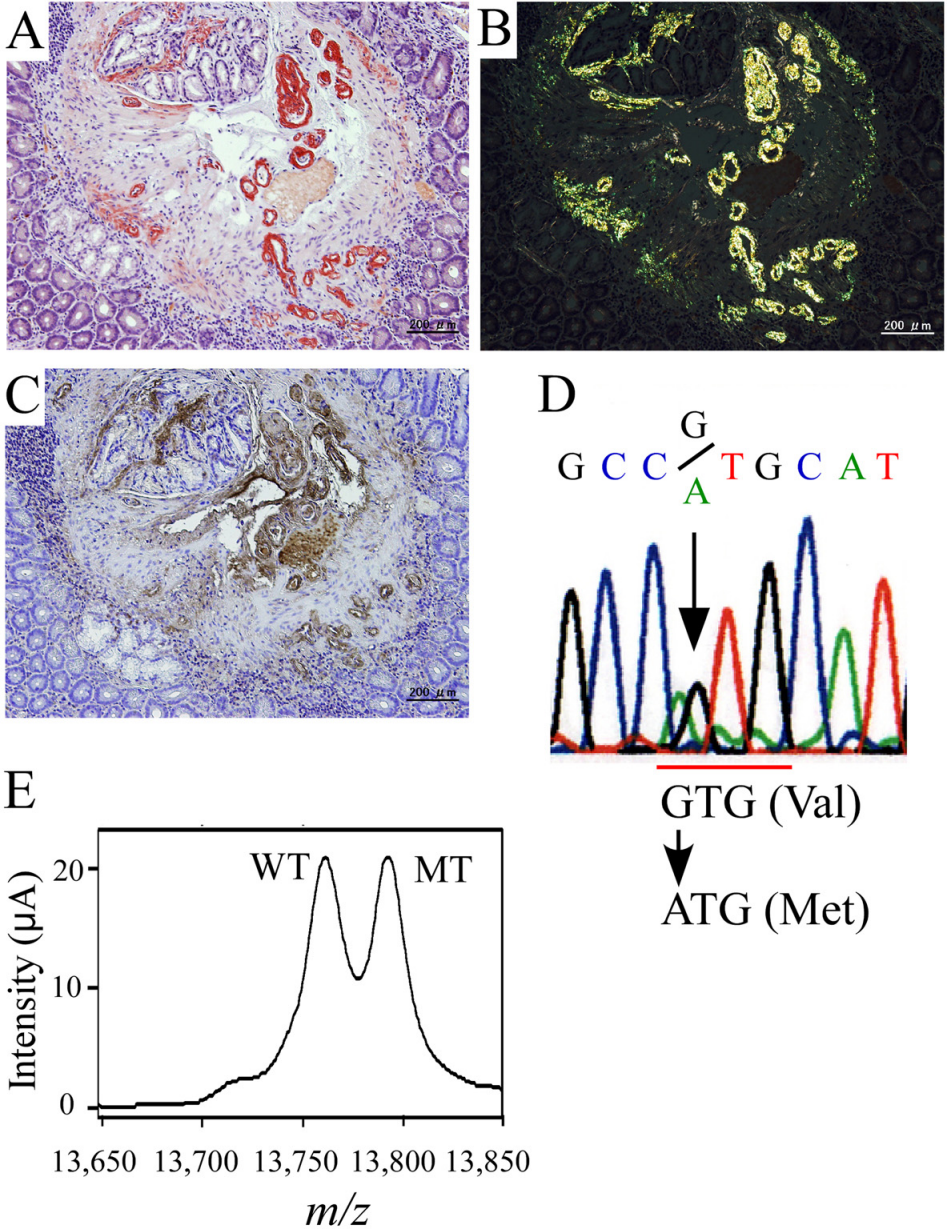
**Diagnostic studies for FAP. (A-C)** Histopathological images of a biopsy specimen of the duodenum obtained from an FAP ATTR Val30Met patient. **(A)** Congo red staining. **(B)** Polarized light. **(C)** Immunohistochemistry with an anti-TTR antibody. **(D)** Sequence analysis of the TTR gene. **(E)** SELDI-TOF MS analyses of serum TTR from an FAP ATTR Val30Met patient. The molecular weight of MT TTR is 32 *m*/*z* higher than that of WT TTR.

Effective medical treatment of patients with TTR amyloidosis requires an accurate diagnosis based on various studies and techniques [42], such as histopathology, genetic testing, and mass spectrometry (Figure 4). Although diagnosis in the early stage of FAP is absolutely imperative for proper treatment, the diagnosis is sometimes delayed, especially in patients without a clear family history and typical clinical manifestations of FAP [43,44].

Histopathological examinations play a critical role in obtaining direct evidence of amyloid deposits and determining the type of amyloid-causing protein [45]. Systemic amyloidosis including FAP is usually diagnosed on the basis of biopsies of several tissue sites, such as subcutaneous adipose tissue of the abdominal wall [46], GI tract [47,48], and labial salivary gland [49], because biopsies of tissue sites with main clinical symptoms such as peripheral nerves, heart, and kidney are more invasive. Biopsy specimens are subjected to Congo red staining to detect amyloid deposits and are viewed with a microscope under polarized light. In patients with only a small amount of amyloid deposition, a confirmed diagnosis may require repeated biopsies. Most often, immunohistochemical determination of the chemical composition of amyloid is the first step in classifying the type of amyloid: a panel of antibodies recognizing different amyloid types is used to reveal the origin of deposited protein [50,51]. However, misdiagnoses have occurred in some cases, especially when immunohistochemical staining is performed in the absence of standardized antibodies and appropriate positive controls [52]. Extraction of amyloid fibril proteins from frozen and formalin-fixed tissues or from amyloid-containing histopathological tissue sections followed by immunostaining or amino acid sequence analyses is another useful way to characterize amyloid deposits [53,54], especially when immunohistochemical data are negative or inconclusive. A novel rapid test to determine the type of amyloidosis based on tandem mass spectrometric analysis, with specific sampling of clinical biopsy specimens by means of laser microdissection, was recently reported [55,56]. With this method, the authors successfully identified the amyloid proteins and classified the types of amyloidosis including TTR amyloidosis [39,57]. This method may be a useful clinical tool for aiding the accurate typing of amyloidosis.

To obtain accurate results for the TTR mutation in FAP patients or carriers of variant TTR genes, both genetic and proteomic methods should be applied to compensate for the disadvantages and possible pitfalls of each technique (Figure 4). Mass spectrometric analysis allows detection of variant TTRs in serum, because an amino acid substitution results in a change in the molecular weight of TTR circulating in the bloodstream [58-60]. We recently applied surface-enhanced laser desorption/ionization time-of-flight mass spectrometry (SELDI-TOF MS) to rapid detection of variant TTR in a patient’s serum [61]. This technique allows the analysis of a variant form of TTR in a one-step procedure.

### FAP treatments

The sections that follow provide details of conventional and potential treatments of TTR amyloidosis (Figure 1).

#### ***Liver transplantation***

With regard to treatment of FAP, liver transplantation (LT) has reportedly halted the progression of clinical manifestations [62,63]. Exchange of an FAP patient’s diseased liver with a healthy liver causes MT TTR in the body to be replaced by WT TTR, except for cerebrospinal fluid and eyes, into which MT TTR is secreted from the choroid plexus and the retina, respectively, even after LT [64,65]. Since 1990, FAP patients have undergone LT as FAP treatment [66,67]. According to data in the FAP World Transplant Registry [66], approximately 120 orthotopic LTs are performed worldwide each year. LT reportedly prolonged the survival of FAP ATTR Val30Met patients who were carefully selected for the procedure [68,69]. The modified body mass index, disease duration, age, type of TTR mutation, and degree of cardiac involvement are thought to be important prognostic factors for the disease course after LT [62,67,69]. The survival rate of FAP ATTR non-Val30Met patients after LT was reportedly less than that of FAP ATTR Val30Met patients after LT [66,70,71]. LT could be less effective for the patients with ATTR non-Val30Met who have cardiomyopathy or leptomeningeal dominant symptoms.

Our criteria for performing LT for FAP patients were as follows: age younger than 60 years, duration of disease from onset <5 years, creatinine clearance >70 ml/min, modified body mass index >500, no cardiomegaly, and no gait disturbance [72]. The main causes of death in patients after orthotopic LTs involved cardiac problems (24%), sepsis (23%), and liver-related complications (14%) [66,70,71]. Although clinical evaluations indicated that progression of other clinical symptoms such as peripheral neuropathy [73,74], GI symptoms [75], and renal involvement [72,76] usually stopped after LT in FAP ATTR Val30Met patients, other studies suggested that LT failed to prevent progression of cardiac amyloidosis in FAP ATTR Val30Met patients after LT [69,77], with this failure reportedly being due to continued formation of amyloid mainly derived from WT TTR secreted from the transplanted normal liver graft [29,30,78]. However, why WT TTR amyloid deposits, which are usually found in elderly people with SSA, occur in some tissue sites of FAP patients after LT remains to be clarified. It may be that older amyloid deposits formed by MT TTR before LT may act as a nidus, which is well known to enhance polymerization of proteins [79], and that additional WT TTR amyloid fibrils may form because of nucleation-dependent polymerization after LT, although TTR amyloid formation *in vitro* reportedly did not depend on nucleation [80].

Because of the shortage of livers for transplantation to patients with malignant or end-stage liver diseases, the method of using sequential LT with resected livers from FAP patients was developed [81]. Some patients who underwent sequential LT with livers from FAP patients reportedly started to evidence TTR amyloid deposits less than 10 years after the surgery [82-84]. However, we do not know whether all such second recipients will eventually have symptoms of FAP.

#### ***Stabilizers of TTR tetramers: tafamidis and diflunisal***

The working hypothesis of amyloid formation established by a substantial number of studies led to the idea that stabilizing tetrameric TTR would be a promising method to prevent amyloid formation [85]. Tetrameric TTR itself is thought to be nonamyloidogenic, but dissociation of the tetramer into compact non-native monomers with low conformational stability can lead to amyloid fibril formation [86]. Baures *et al*. reported that, on the basis of *in vitro* experiments, various nonsteroidal anti-inflammatory drugs have potential for stabilizing tetrameric TTR [87]. These efforts thoroughly established this possibility of stabilization of the tetrameric form of TTR as a therapeutic strategy.

Two drugs—tafamidis, which is a novel TTR stabilizer, and diflunisal, which is a nonsteroidal anti-inflammatory drug developed in 1971 and can stabilize TTR tetramers—are undergoing clinical development throughout the world. Tafamidis, a potent and selective stabilizer of tetrameric TTR, has been approved in Europe and Japan for treatment of adult FAP patients with early symptomatic polyneuropathy to delay neurological impairment [88,89]. In the clinical trial, patients treated with tafamidis had less neurological deterioration than patients who began tafamidis later; they had some preservation of function as measured by the Neuropathy Impairment Score-Lower Limb [88,89]. Recently, Berk et al. also reported that diflunisal reduced the rate of progression of neurological impairment and preserved quality of life [90]. Therapeutic effects of those TTR stabilizers on long-term outcomes, cardiac functions and ophthalmic symptom remain to be elucidated. Moreover, a number of structurally diverse small molecules that bind to TTR, increase its stability, and thereafter inhibit amyloid fibrillogenesis have been tested.

#### ***Gene therapies to suppress TTR expression***

Knowledge gained by using LT as therapy for FAP suggested that inhibition of amyloidogenic TTR may prevent progression of the disease. Antisense methods and small interfering RNAs (siRNAs) are effective gene-silencing tools. The antisense oligonucleotides (ASO), which were recognized as a therapeutic tool in the 1970s, cause enzymatic degradation of mRNA. RNA interference, first discovered in *Caenorhabditis elegans*, is sequence-specific post-transcriptional gene silencing [91]. Specific gene expression has also been inhibited by RNA interference in mammalian cells by skipping the Dicer step [92]. These methods should be powerful tools for FAP gene therapy.

Early studies in this field aimed to selectively inhibit production of MT TTR. Different kinds of siRNA selectively silenced TTR gene expression both *in vitro* and *in vivo*[93]. Recently developed siRNA and ASO therapies, however, inhibit both MT TTR and WT TTR [94,95], because WT TTR also contributes to the formation of amyloid in FAP, especially after LT. Benson *et al*. demonstrated that ASO suppressed TTR mRNA levels in the liver and in the choroid plexus of the brain [96,97]. Researchers completed a phase I study evaluating the safety and activity of ASO in healthy volunteers [95]. ASO reduced plasma TTR protein levels up to 80% without causing clinically significant adverse reactions. A phase II/III study evaluating the efficacy of ASO in patients with FAP is ongoing in 2014. Also, a phase I clinical study with siRNA for FAP was completed [94], and a phase II/III study is ongoing in 2014.

#### ***Retinal laser photocoagulation***

Even after LT, ocular complications have reportedly persisted and worsened, because RPE cells of the eye continued to synthesize MT TTR in FAP patients [64,98]. To suppress TTR synthesis and ocular symptoms, we have evaluated retinal laser photocoagulation. This operation, which is commonly used to treat many retinal diseases, damages the retinal pigment epithelium, the main location of synthesis of ATTR in ocular tissues. To date, we discovered that retinal laser photocoagulation clearly prevented progression of amyloid deposition in the vitreous and on the retinal surface in certain FAP patients, without causing any adverse effects [99]. Retinal laser photocoagulation may thus be a new procedure for mitigating ocular manifestations in FAP patients.

#### ***4′-Iodo-4′-deoxydoxorubicin, doxycycline, tauroursodeoxycholic acid, and cyclodextrin***

Other candidate therapeutic compounds, including 4′-iodo-4′-deoxydoxorubicin (IDOX), doxycycline, tauroursodeoxycholic acid (TUDCA), and cyclodextrin (CyD), have also been studied.

Merlini *et al*. first reported IDOX as an agent that would bind to amyloid fibrils found in five different types of amyloidosis [100]. Sebastiao *et al*., in an *in vitro* study, noted the interaction of IDOX and ATTR Leu55Pro and reported the rapid dissociation of monoclinic ATTR Leu55Pro crystals soaked with IDOX [101].

Doxycycline influences many functions of mammalian cells such as proliferation, migration, apoptosis, and matrix remodeling [102]. Cardoso and colleagues investigated the effects of doxycycline treatment *in vivo* by using ATTR Val30Met transgenic mice [103,104].

TUDCA is a unique natural compound that is a potent antiapoptotic and antioxidant agent, as it reduces cytotoxicity in a number of neurodegenerative diseases. Macedo *et al*. studied the possible therapeutic application of TUDCA in FAP [105] and found that TUDCA treatment significantly decreased the amount of TTR toxic aggregates.

CyDs are cyclic oligosaccharides composed of 6–8 glucose units [106]. Because CyDs contain a central hydrophobic cavity, which can serve as an inclusion site for hydrophobic molecules, CyDs are now used as multifunctional drug carriers [107]. Jono *et al*. reported that 6-*O*-α-(4-*O*-α-d-glucuronyl)-d-glucosyl-β-CyD (GUG-β-CyD), a branched β-CyD derivative, may inhibit TTR misfolding by stabilizing the conformation of TTR by means of interacting with hydrophobic amino acids, especially the Trp residue of TTR, which thereby suppresses TTR amyloid formation [108]. CyDs are safe and already widely used in many fields, especially pharmaceuticals, so GUG-β-CyD may become a curative drug for TTR amyloidosis.

#### ***Gene conversion therapy***

As described above, LT was originally suggested as a treatment that would halt production of variant TTR in the liver. Most FAP symptoms do not progress after LT, when the MT TTR gene is replaced by the WT TTR gene in the liver. This finding led to the suggestion that gene therapy to correct the TTR gene mutation may ameliorate the clinical symptoms of FAP. Nakamura *et al*. demonstrated gene conversion by single-stranded oligonucleotides in rabbit eyes expressing rabbit WT TTR and in transgenic murine liver in which the intrinsic WT TTR gene was replaced by a TTR Val30Met gene [109].

#### ***Immunotherapies***

Immunotherapies are also major candidates for TTR amyloidosis treatment. Gustavsson *et al*. used various antigenic mapping methods to find out whether major antigenic sites differed for normal TTR, ATTR, and *in situ* amyloid fibrils [110]. Their data suggested that antigenic sites on normal plasma TTR included the AB and CD loops, with the associated amino acid sequences occurring on the outside of the TTR molecule. An antiserum against β-strand H (anti-TTR115-124), which establishes the dimer’s monomer-to-monomer interaction areas, reacted with only ATTR in amyloid fibrils, not with normal TTR in plasma. These findings suggested an altered TTR configuration in amyloid fibrils compared with the TTR configuration in plasma. Thus, anti-TTR115-124, which seems to be amyloid specific, may be valuable as a probe and in antibody therapies.

An MT TTR Tyr78Phe that was designed to destabilize the native structure of TTR tetramer has exposed a cryptotope recognized by a monoclonal antibody that reacts only with amyloid fibrils or with highly amyloidogenic MTs that present the amyloid fold [111]. Terazaki *et al*. demonstrated that immunization with TTR Tyr78Phe effectively reduced TTR deposition and cleared amyloid deposits in an FAP rodent model transgenic for human TTR Val30Met with amyloid deposition in the GI tract [112]. This therapy may be applied to FAP ATTR Val30Met patients with amyloid deposition in tissues. In addition, this treatment may be useful for vaccination of healthy variant TTR gene carriers to prevent TTR amyloid deposition in tissues.

Serum amyloid P component (SAP) is a major component of amyloid deposits in all types of amyloidoses. Recently, Bodin, et al. investigated therapeutic effects of anti-human SAP antibodies on AA amyloid deposition using a human SAP transgenic mouse model [113]. The antibodies removed AA amyloid deposits in the mouse model. This antibody therapy might be applicable to FAP.

## Conclusions

Although LT and TTR stabilizers became practical treatments for FAP, there remain many clinical issues we have to improve. Several clinical trials using other new methods are ongoing. These novel therapies may prove to prevent progression of FAP.

## Abbreviations

ASO: Antisense oligonucleotides; ATTR: Amyloidogenic TTR; FAP: Familial amyloidotic polyneuropathy; GI: Gastrointestinal; GUG-β-CyD: 6-*O*-α-(4-*O*-α-d-glucuronyl)-d-glucosyl-β-CyD; IDOX: 4′-iodo-4′-deoxydoxorubicin; LT: Liver transplantation; MT: Mutant; RPE: Retinal pigment epithelial; SELDI-TOF MS: Surface-enhanced laser desorption/ionization time-of-flight mass spectrometry; siRNAs: Small interfering RNAs; SSA: Senile systemic amyloidosis; TTR: Transthyretin; TUDCA: Tauroursodeoxycholic acid; WT: Wild-type.

## Competing interests

The authors declare that they have no competing interests.

## Authors’ contributions

MU and YA drafted and revised the manuscript. Both authors read and approved the final manuscript.
